# The predictive roles of self-compassion, perceived social support, and psychological flexibility in early maladaptive schemas among college students: an exploration based on latent profile analysis

**DOI:** 10.3389/fpsyg.2025.1619308

**Published:** 2025-08-29

**Authors:** Sicen Zhang, Quandong Liu, Menglu Jia, Qiuying Zhang, Lixia Zhang

**Affiliations:** ^1^School of Public Health, Shaanxi University of Chinese Medicine, Xianyang, China; ^2^Henan Children’s Hospital Zhengzhou Children’s Hospital, Zhengzhou, China; ^3^Beijing Huilongguan Hospital, Peking University, Beijing, China

**Keywords:** early maladaptive schemas, psychological flexibility, self-compassion, perceived social support, college and university students

## Abstract

**Purpose:**

This study used latent profile analysis (LPA) to identify subgroups of early maladaptive schemas (EMSs) among college students based on the five core schema domains, and then investigated how these profiles related to self-compassion, perceived social support, and psychological flexibility.

**Methods:**

A total of 1,184 college students from universities in Northwest China were selected using cluster sampling (47.3% male, 52.7% female; 58.1% freshmen, 21.4% sophomores, 12.8% juniors, and 7.7% seniors). Participants completed a cross-sectional survey including the Short Form of the Young Schema Questionnaire, the Self-Compassion Scale, the Perceived Social Support Scale, and the Avoidance and Fusion Questionnaire (Short Form).

**Results:**

LPA identified three different EMSs profiles: low, moderate, and high. The high group scored significantly higher in disconnection and rejection, impaired autonomy and performance, impaired limits, other-directedness and over-vigilance and inhibition compared with the other two groups, while the low group demonstrated the lowest scores across all domains. Additionally, degrees of self-compassion, perceived social support, and psychological flexibility differed considerably between profiles. Multinomial logistic regression indicated that gender, grade level, self-compassion, perceived social support, and psychological flexibility significantly predicted profile membership in the expected directions.

**Conclusion:**

The study revealed clear variations in EMSs among college students, yielding three distinct profiles. The findings support the hypothesis, and provide a theoretical basis for developing targeted psychological interventions aimed at enhancing self-compassion, strengthening social support, and improving psychological flexibility.

## 1 Introduction

### 1.1 Early maladaptive schemas and their psychological impact

With increasing social competition and the accelerated pace of university life, the mental health of college students has received growing attention. Many of these psychological difficulties have developmental roots traceable to childhood experiences. Young’s schema therapy theory provides a crucial framework for understanding the formation mechanisms of individual psychological problems (Young et al., 2006). Early maladaptive schemas (EMSs), initially conceptualized by Young, refer to enduring and pervasive cognitive-affective patterns that emerge during childhood, often as a consequence of unmet emotional needs (Riso et al., 2006). These schemas are categorized into 18 distinct types across five domains: disconnection and rejection (DR), impaired autonomy and performance (IAP), impaired limits (IL), other-directedness (OD), and over-vigilance and inhibition (OVI) (Young et al., 2006). EMSs significantly influence individuals’ cognitive processing (Bach et al., 2018), emotional regulation, and interpersonal behavior across the lifespan (Ghosh and Gilboa, 2014). Once established, EMSs become reinforced through biased cognitive mechanismsand maladaptive coping strategies (Mairet et al., 2014), often culminating in long-term psychological distress (Janovsky et al., 2020) and the development of dysfunctional behavior patterns (Lobbestael and Arntz, 2012). For college students, EMSs can become significant obstacles to academic adjustment, emotional development, and career planning (Cámara and Calvete, 2012). Research has shown a strong correlation between EMSs and negative psychological states such as anxiety and depression (Early, 2023), as well as psychiatric disorders (Thimm and Chang, 2022). Longitudinal studies on large samples have demonstrated that adolescents with high scores in the disconnection and rejection, impaired autonomy and performance, and other-directedness schema domains are significantly more likely to develop symptoms of social anxiety and depression later in life (Calvete et al., 2015), strongly supporting the negative impact of EMSs on mental health. Individuals who can effectively mobilize positive psychological resources and adopt adaptive coping strategies in response to schema-related stressors may be able to avoid developing pathological behavior patterns (Ünal, 2012)^.^ Although much attention has been paid to these negative outcomes, it is equally important to examine how individuals positively respond to EMSs-related challenges. This perspective aligns with recent developments in positive psychology, which emphasize resilience and internal strengths.

### 1.2 Positive psychological resources in relation to EMSs

To date, limited research has systematically examined the associations between EMSs and positive psychological factors. A substantial body of research has predominantly focused on their impact on negative emotions and mental disorders, overlooking the potential positive psychological resources individuals may exhibit when confronted with EMSs. Given the potential role of these positive factors in interventions, investigating their associations with e EMSs not only contributes to a more comprehensive understanding of individual adaptation mechanisms but also provides novel perspectives and intervention strategies for promoting mental health among university students.

The rise of positive psychology has provided a new perspective on examining EMSs. Martin Seligman proposed positive psychology, which tries to examine the elements that lead to human well-being and psychological health (Seligman et al., 2005). Unlike traditional problem-focused psychological research (Yildirim et al., 2023), positive psychology emphasizes the exploration of individual potential (Yıldırım and Green, 2024) and the possibilities for personal growth (Yıldırım and Aziz, 2023). To further substantiate this perspective within a theoretical framework, the present study incorporates self-determination theory, which posits that innate psychological needs—autonomy, competence, and relatedness—are essential for fostering intrinsic motivation, facilitating personal growth, and maintaining psychological well-being (Deci, 2010). Self-compassion, perceived social support, and psychological flexibility link to the three main psychological needs: autonomy, relatedness, and competence. These three components interact to create an internal motivating system (Green et al., 2024) that promotes good psychological development (Yıldırım et al., 2024) and serves as a solid foundation for overall well-being (Yildirim et al., 2024), social adaption (Öztekin et al., 2025), and personal growth (Yıldırım et al., 2024). Self-compassion refers to an individual’s ability to treat themselves with care, acceptance, and understanding in the face of difficulties or negative emotions rather than engaging in self-criticism or harsh judgment (Arslan et al., 2021). It plays an essential role in fulfilling the need for competence (Neff, 2003). Studies have shown a significant negative correlation between self-compassion and EMSs (Ezzati et al., 2024). According to Thimm’s research, self-compassion allows people to stay calm and sensible in tough situations, mobilize internal resources, and actively deal with obstacles (Thimm, 2017). Self-compassion prevents the formation and maintenance of EMSs by minimizing self-criticism and negative thinking, encouraging a sense of competence. Perceived social support refers to an individual’s perception of support from family, friends, and society. It not only provides psychological security but also enhances a sense of belonging and helps individuals cope with stress (Cohen and Wills, 1985). Shelton et al. found that perceived social support is significantly positively correlated with psychological well-being (Shelton et al., 2017). By fulfilling the need for relatedness, perceived social support effectively alleviates anxiety, depression, and other negative emotions (Ryan and Solky, 1996) and plays a role in restructuring maladaptive schemas (Shahbeik et al., 2023). Psychological flexibility refers to an individual’s ability to accept negative emotions and stress while engaging in adaptive behaviors (Harris, 2019). Nelson et al. discovered that increased psychological flexibility is associated with enhanced neuroplasticity in the prefrontal cortex (Nelson and Guyer, 2011), which is responsible for higher-order cognitive functioning and behavioral regulation (Dunkley et al., 2015). Individuals may efficiently integrate internal and external information, allowing them to make autonomous decisions and adjust their behavior. According to Borjali et al., individuals with higher psychological flexibility can mitigate the negative impact of EMSs on anxiety, potentially by modifying cognitive patterns, accepting emotions, and adjusting behaviors to break free from rigid schemas (Borjali et al., 2016). This also suggests a strong inverse relationship between EMSs and psychological flexibility. However, previous research on EMSs has primarily focused on single-variable influences, separately examining the effects of perceived social support, self-compassion, and psychological flexibility on EMSs. Such a variable-centered approach may fail to capture the dynamic interplay among positive psychological resources and may insufficiently account for the heterogeneity in EMS manifestations across individuals. Accordingly, a more holistic, person-centered analytical framework is warranted to identify meaningful latent subgroups and to examine how combinations of positive traits jointly predict schema configurations.

### 1.3 Heterogeneity of EMSs and the need for a person-centered approach

The heterogeneity of EMSs arises from the interaction between innate temperament and repeated exposure to adverse experiences during childhood (Young et al., 2006). Based on Pavlov’s theory of higher nervous activity, individuals differ in the strength, balance, and flexibility of their neural processes, fundamentally shaping their perception, processing, and response to environmental stimuli (Pavlov, 2020). Individuals with high neuroreactivity, particularly those with heightened amygdala sensitivity, are more responsive to negative emotional stimuli (Palamarchuk and Vaillancourt, 2021). When exposed to adverse childhood experiences, they are more likely to exhibit strong and persistent neurophysiological reactions, encoding these experiences as threats and subsequently internalizing them as schemas (Mairet et al., 2014). In contrast, individuals with lower neuroreactivity may exhibit weaker responses to similar experiences and are less likely to develop rigid schemas (Halvorsen et al., 2009). Interpersonal experiences, particularly early caregiver relationships, further contribute to EMSs development. Attachment theory provides a complementary lens in this regard. Attachment theory posits that early attachment relationships with primary caregivers play a crucial role in psychological development (Goldberg et al., 1995). Different attachment styles contribute to variations in cognition, emotion, and behavior, leading to highly heterogeneous EMSs patterns. Research has shown a significant positive correlation between insecure attachment and EMSs, while secure attachment is negatively correlated with EMSs. Compared to avoidant attachment, anxious attachment is more strongly associated with the disconnection and rejection and other-directedness schema domains (Karantzas et al., 2023). However, traditional variable-centered research methods, which classify individuals based on predetermined threshold values, fail to effectively capture the interaction effects between multiple variables or determine the distribution of different latent subgroups within a population.

Latent Profile Analysis (LPA) is a person-centered statistical approach used to identify hidden subgroups within a dataset based on patterns observed across multiple variables (Tein et al., 2013). Unlike traditional group comparison methods, LPA does not rely on predefined categories; instead, it employs data-driven techniques to uncover latent classes and assigns individuals probabilistically to these classes (Williams and Kibowski, 2016). This makes LPA particularly suitable for examining heterogeneous schema profiles and investigating how positive psychological traits differentiate among subgroups.

### 1.4 The present study

Based on the above theoretical and empirical background, this study aims to integrate self-compassion, perceived social support, and psychological flexibility into a person-centered analytical framework using LPA to identify distinct EMSs profiles among college students. Specifically, this section expands the rationale by: (Young et al., 2006) summarizing key gaps in prior research, such as the lack of integrated, multi-trait person-centered studies on EMSs; (Riso et al., 2006) highlighting the importance of combining self-compassion, perceived social support, and psychological flexibility in distinguishing latent profiles; and (Bach et al., 2018) linking the study design to schema theory, positive psychology, and self-determination theory. The objectives of this study are to identify latent EMSs profiles among college students using LPA, to examine how self-compassion, perceived social support, and psychological flexibility differ across these profiles, and to assess whether these traits predict EMS profile membership.

Based on theoretical considerations and prior empirical findings, the following hypotheses are proposed:

H1: Higher levels of self-compassion will be associated with increased likelihood of membership in lower EMSs profile classes.

H2: Higher levels of perceived social support will be associated with increased likelihood of membership in lower EMSs profile classes.

H3: Higher levels of psychological flexibility will be associated with increased likelihood of membership in lower EMSs profile classes.

## 2 Method

### 2.1 Participants

Data for this study were acquired using “Wenjuanxing,” a popular online questionnaire platform in China. Before participating, all participants gave their informed consent. The study was authorized by the ethical committee of the researchers’ university. The process began with identifying the target universities and majors, followed by contacting student counselors or course instructors to request their help. The survey was carried out on-site, according to a predetermined schedule. Before completing the questionnaire, participants were informed about the study’s purpose, survey instructions, and any necessary precautions. The concepts of voluntary participation and confidentiality were emphasized, and participants were told to respond truthfully based on their own experiences. The questionnaire and informed permission form were issued jointly, and the completion duration was around 20–25 min. A total of 1,327 questionnaires were distributed. After excluding 143 invalid responses due to failed lie-detection items or abnormally short completion times, 1,184 valid responses were retained (response rate = 89.2%). Demographic characteristics are presented in Table 1.

**Table 1:** Demographic characteristics.

Variable	Category	*n*	%
Gender	Male	560	47.3
	Female	624	52.7
Grade	Freshman	688	58.1
	Sophomore	253	21.4
	Junior	152	12.8
	Senior	91	7.7
Only-child status	Yes	344	29.1
	No	840	70.9
Residence	Urban	496	41.9
	Rural	688	58.1

### 2.2 Measures

#### 2.2.1 Young Schema questionnaire (short form)

Early maladaptive schemas were assessed using the Young Schema Questionnaire-Short Form (YSQ-SF), which was created by Young and translated into Chinese by Li-Xia et al. (2012). The scale has 75 items that are scored on a 6-point Likert scale ranging from 1 (totally false) to 6 (entirely true), with higher scores suggesting a higher level of maladaptive schemas. The questionnaire has five subscales: disconnection and rejection, impaired autonomy and performance, impaired limitations, other-directedness, and over-vigilance and inhibition, with no reverse-coded items. The Cronbach’s α for the subscales ranged from 0.70 to 0.92.

#### 2.2.2 Avoidance and fusion questionnaire (short form)

Greco et al. developed the Avoidance and Fusion Questionnaire for Youth (AFQ-Y8, Short Form), which Chen et al. (2019) converted into Chinese to assess psychological flexibility. The scale comprises eight items and measures a single factor structure; there are no reverse-coded items. Participants rate each topic on a 5-point Likert scale, with higher scores indicating less psychological flexibility and more cognitive rigidity. The Cronbach’s α for this scale was 0.78.

#### 2.2.3 Self-compassion scale

Self-compassion was measured using the Self-Compassion Scale (SCS), which was created by Neff and translated into Chinese by Gong et al. (2014). The scale consists of 12 items that assess three dimensions: self-kindness, common humanity, and awareness. Each item is scored on a 5-point Likert scale ranging from 1 (nearly never) to 5 (almost always), with higher scores indicating more self-compassion. Items 2, 4, 5, 8, and 11 are scored in reverse. The Cronbach’s α for the scale was 0.77.

#### 2.2.4 Perceived social support scale

The Perceived Social Support Scale (PSSS), created by Zimet et al. (1988) and translated into Chinese, was used to assess perceived social support (Zimet et al., 1988). The scale consists of 12 items, each scored on a 7-point Likert scale ranging from 1 (strongly disagree) to 7 (strongly agree), with higher scores indicating better perceived social support. The measure has three subscales that evaluate assistance from family, friends, and other social sources. The Cronbach’s α for this scale was 0.896.

### 2.3 Statistical analysis

SPSS 27.0 was used for data preprocessing and analysis, including common method bias testing, descriptive statistics, correlation analysis, and subsequent logistic regression analysis. Latent profile analysis (LPA) was conducted using Mplus 8.3. Model fit was evaluated using Akaike Information Criterion (AIC), Bayesian Information Criterion (BIC), sample-size adjusted BIC (aBIC), entropy, Lo–Mendell–Rubin likelihood ratio test (LMR), and bootstrap likelihood ratio test (BLRT). The optimal number of profiles was determined based on lower AIC, BIC, and aBIC values, higher entropy values, and significant LMR and BLRT results. Differences in self-compassion, perceived social support, and psychological flexibility between profiles were examined using analysis of variance (ANOVA) with Bonferroni correction for multiple comparisons. Multinomial logistic regression analysis was performed to test whether demographic and psychological variables predicted EMSs profile membership.

## 3 Results

### 3.1 Common method bias analysis

As we collected the data via questionnaires, common method bias may exist. Thus, we conducted the Harman single factor test through confirmatory factor analysis. The results showed that there were 21 eigenvalues greater than 1 without rotation, and the mutation rate interpretation of the first factor was 25.388%, which was less than the critical value of 40%, thereby indicating that no serious problem of common method bias existed in our study.

### 3.2 Latent profile analysis

Latent class analysis was performed using Mplus 8.3. The mean scores of the five schema domains of EMSs were included in the model. Models with 2 to 6 latent classes were tested sequentially, and model fit indices were compared to determine the optimal classification. Model fit was evaluated using AIC, BIC, aBIC, along with entropy values. Additionally, the Lo–Mendell–Rubin Likelihood Ratio Test (LMR-LRT) and BLRT were used to assess model fit.

Smaller values of AIC, BIC, and aBIC indicate better model fit. The entropy value ranges from 0 to 1, with values closer to 1 indicating higher classification accuracy. Significant *p*-values for LMR-LRT and BLRT suggest that the k-class model is significantly better than the k-1 class model (Qiu, 2008). The model fit results are shown in Table 2.

**Table 2:** Latent profile analysis model information.

Class	2	3	4	5	6
AIC	11384.856	10489.909	10211.792	10094.024	9964.736
BIC	11466.082	10601.595	10353.938	10266.631	10167.803
ABIC	11415.261	10531.715	10,265	10158.634	10040.748
ENTROPY	0.962	0.93	0.896	0.871	0.889
MLR-LRT	0	0	0.0023	0.0003	0.0429
BLRT	0	0	0	0	0

**Figure 1:**
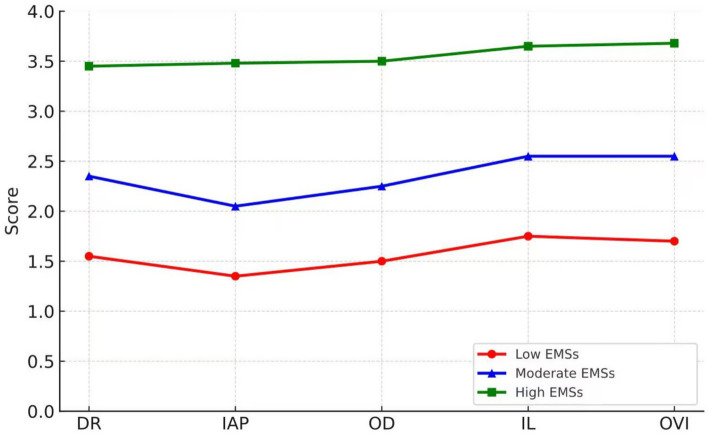
Profile analysis results.

Model fit information suggests that as the number of categories increases, the AIC, BIC, and aBIC values show a decreasing trend. The LMR-LRT and BLRT indices are significant across all category models. Considering the entropy index, the entropy values for both the two-category and three-category models exceed 0.9. Taking into account category probabilities and model interpretability, the three-category model was ultimately selected. The model is shown in Figure 1.

The three latent categories were named as follows: the first category was named the low EMSs, consisting of 210 individuals (17.9%); the second category was named the moderate EMSs, consisting of 349 individuals (29.2%); and the third category was named the high EMSs, consisting of 625 individuals (52.9%). The descriptive statistics for each category and the results of the difference tests for each schema domain are presented in Table 3.

**Table 3:** Descriptive statistics and difference testing.

Class	DR	IAP	OD	IL	OI
Low EMSs	1.54 ± 0.43	1.20 ± 0.23	1.50 ± 0.41	1.85 ± 0.65	1.78 ± 0.56
Moderate EMSs	2.37 ± 0.44	1.99 ± 0.42	2.32 ± 0.47	2.67 ± 0.61	2.71 ± 0.60
High EMS	3.42 ± 0.40	3.48 ± 0.42	3.50 ± 0.49	3.63 ± 0.55	3.65 ± 0.56
F	1852.28***	3243.96***	1673.56***	805.15***	928.17***
Post hoc LSD	3>2>1	3>2>1	3>2>1	3>2>1	3>2>1

*p < 0.05, **p < 0.01, ***p < 0.001.

The descriptive statistics indicate that the high EMSs scored approximately 3.5 across all five schema domains. In contrast, for the low and moderate EMSs, the scores for autonomy and incompetence were lower than those in other schema domains. The analysis of variance (ANOVA) results revealed significant differences among the three groups in all five schema domains. *Post hoc* comparisons showed that the differences in EMSs scores between any two groups were statistically significant, indicating clear heterogeneity in classification. It is important to note that the labels “low,” “moderate,” and “high EMSs” assigned to the latent profiles in this study were applied *post hoc*, based on the relative mean levels of EMSs scores across the three identified profiles, rather than being derived from predefined hypotheses. These labels were introduced primarily for interpretive convenience and to facilitate the communication of the results. These labels are based on the empirical, data-driven results of the latent profile analysis, rather than any prior assumptions or theoretical expectations. This approach allows for a more flexible and descriptive categorization of the latent profiles, but caution should be exercised when interpreting these labels in clinical or theoretical contexts.

### 3.3 Exploration of category characteristics

Further difference tests were conducted to examine variations in related variables and demographic variables across the three schema groups. One-way ANOVA was used for continuous variables, while the chi-square test was applied to categorical variables. The results are presented in Table 4.

**Table 4:** Analysis of variance (ANOVA).

Variable	MS	F	Post hoc	η^2^
perceived social support	72.45	125.01***	3>2>1	0.18
self-compassion	26.15	122.71***	3>2>1	0.17
psychological flexibility	26.16	95.13***	3>2>1	0.14

*p < 0.05, **p < 0.01, ***p < 0.001.

The ANOVA results indicate that perceived social support, self-compassion, and psychological flexibility significantly differ among the three schema groups. Bonferroni correction was applied to control for Type I error in the pairwise comparisons. Post hoc comparisons further revealed that the differences in scores for these three variables between any two groups were statistically significant.

The chi-square test results indicate that grade, gender, only-child status, and place of origin all show statistically significant differences among the three groups. Specifically, differences were highly significant for grade (χ^2^ = 454.81, *p* < 0.001) and gender (χ^2^ = 378.18, p < 0.001), and significant for only-child status (χ^2^ = 6.86, *p* < 0.05) and place of origin (χ^2^ = 8.66, p < 0.05), as shown in Table 5.

**Table 5:** Chi-square test.

Variable	χ^2^
Grade	454.81***
Gender	378.18***
Only-child status	6.86*
Place of origin	8.66*

To further explore the influencing factors of each latent class, a multinomial logistic regression analysis was conducted using the significant variables from the difference analysis. The results of the logistic regression analysis are presented in Table 6.

**Table 6:** Multinomial logistic regression analysis.

Class	Moderate EMSs VS low EMSs				High EMSs VS low EMSs				High EMSsVS moderate EMSs			
Variable	B	OR	*p*	95% CIs	B	OR	*p*	95% CIs	B	OR	*p*	95% CIs
Male	−0.43	0.65	0.10	(0.39, 1.09)	−1.76	0.17	0.000	(0.1, 0.3)	−1.33	0.27	0.000	(0.18, 0.4)
Female	0	.	.	.	0	.	.	.	0	.	.	.
Freshmen	0.49	1.63	0.44	(0.47, 5.74)	−2.24	0.11	0.000	(0.04, 0.33)	−2.73	0.07	0.000	(0.03, 0.16)
Sophomores	0.04	1.04	0.95	(0.26, 4.25)	0.28	1.32	0.65	(0.4, 4.37)	0.23	1.26	0.63	(0.49, 3.25)
Juniors	1.22	3.37	0.24	(0.45, 25.18)	1.82	6.17	0.05	(1.02, 37.46)	0.60	1.83	0.29	(0.6, 5.57)
Seniors	0	.	.	.	0	.	.	.	0	.	.	.
Only children	−0.17	0.85	0.48	(0.53, 1.35)	−0.12	0.89	0.66	(0.52, 1.52)	0.05	1.05	0.82	(0.7, 1.59)
Non-only children	0	.	.	.	0	.	.	.	0	.	.	.
Urban students	−0.02	0.98	0.92	(0.65, 1.48)	0.10	1.11	0.69	(0.68, 1.79)	0.12	1.13	0.52	(0.78, 1.64)
Rural students	0	.	.	.	0	.	.	.	0	.	.	.
Psychological flexibility	1.17	3.22	0.000	(2.15, 4.83)	2.08	8.04	0.000	(4.91, 13.17)	0.92	2.50	0.000	(1.69, 3.69)
Perceived social support	−0.57	0.56	0.000	(0.44, 0.72)	−1.12	0.33	0.000	(0.24, 0.44)	−0.55	0.58	0.000	(0.45, 0.74)
Self-compassion	−0.79	0.45	0.001	(0.29, 0.71)	−2.12	0.12	0.000	(0.07, 0.22)	−1.33	0.27	0.000	(0.17, 0.43)

To avoid potential multicollinearity issues, a collinearity check was conducted for the main predictors prior to the logistic regression analysis. The variance inflation factor (VIF), calculated through linear regression, was 1.171, well below the commonly accepted thresholds for multicollinearity concerns (VIF > 5 or 10 (Hair et al., 2019)). Therefore, multicollinearity was not considered a serious issue in this study. Logistic regression results indicate that psychological rigidity is a risk factor for EMSs. Compared to the low EMSs, for individuals with higher levels of psychological rigidity, the probability of belonging to the moderate EMSs increases 3.22 times, and the probability of belonging to the high EMSs increases 8.04 times. Compared to the moderate EMSs, the probability of belonging to the high EMSs increases 2.50 times. Perceived social support and self-compassion are protective factors for EMSs. Compared to the low EMSs, for individuals with higher levels of perceived social support and self-compassion, the probability of belonging to the moderate EMSs decreases by 0.56 times and 0.45 times, respectively, and the probability of belonging to the high EMSs group decreases by 0.33 times and 0.12 times, respectively. Compared to the moderate EMSs, the probability of belonging to the high EMSs decreases by 0.58 times and 0.27 times, respectively. As for demographic variables, females (with males as the reference group) are more likely to belong to the high EMSs. Compared to the low EMSs, the probability increases by 0.17 times, and compared to the moderate EMSs the probability increases by 0.27 times. Seniors (with seniors as the reference group) are more likely than freshmen to belong to the high EMSs (OR = 0.11, *p* < 0.05) and the moderate EMSs (OR = 0.07, p < 0.05). Whether being an only child or the place of origin had no effect on the classification results.

## 4 Discussion

### 4.1 Latent profile characteristics and heterogeneity of EMS s in college students

This study used latent profile analysis to identify three distinct categories of EMSs among college students: low EMSs, moderate EMSs, and high EMSs. The model fit indices suggested a good model fit, indicating significant differences between the profile types. The low EMSs accounted for the smallest proportion, and these students may have experienced a relatively positive developmental environment in childhood, enabling them to cope more effectively with environmental changes and interpersonal stress. The moderate EMSs showed scores for each dimension and total score that fell between the other two groups. This group may face certain psychological and social adaptation challenges and may require appropriate psychological support and optimization of the social environment to enhance their adaptability (Başer Baykal and Erden Çınar, 2023). Young et al. also suggested that EMSs can have varying negative impacts on individuals’ interpersonal relationships and daily lives (Young et al., 2006). Compared to earlier models, such as the traditional 18-schema framework, our three-profile solution offers a more refined and efficient classification of EMSs, emphasizing broad domains of maladaptive behavioral patterns rather than isolating individual schemas. This approach provides a more pragmatic understanding of EMSs heterogeneity, with direct applicability to clinical practice. The three-profile model effectively addresses the complexity inherent in dealing with the 18 individual EMSs types, a limitation identified in previous studies (Young et al., 2006). By focusing on broader categories rather than fragmented, individual schemas, this model enhances clinical efficiency and offers a more streamlined framework for assessment and intervention. The high EMSs was the most prevalent among college students. They scored significantly higher than the low EMSs and moderate EMSs in all five EMSs dimensions and total scores, particularly in the dimensions of impaired limits, over vigilance and inhibition, which had the highest scores. Impaired limits refer to a lack of internal boundaries, difficulty assuming responsibility for others, and challenges in respecting others’ rights, collaborating, honoring commitments, and achieving long-term goals. Over vigilance and inhibition refer to suppressing spontaneous emotions, impulses, and choices, or adhering to overly stringent internalized standards and expectations related to achievement and moral behavior, often at the expense of pleasure, self-expression, relaxation, close relationships, and health (Young et al., 2006). This might cause college students to focus too much on internal discomfort and the urge for control, ignoring the actual responsibilities of life and education. According to Bazargani et al.’s findings, insufficient restriction has a bigger detrimental impact than other schema areas, followed by hypervigilance and suppression (Bazargani et al., 2023). These findings indicate that university mental health educators should prioritize developing students’ senses of responsibility and self-control during treatments. This can be accomplished by providing group counseling courses on related issues, developing responsibility and teamwork training activities, and coaching students in recognizing and correcting behavioral biases. Furthermore, universities can offer psychological counseling services to assist students in accepting their feelings and needs, adjusting accomplishment and moral expectations appropriately, and encouraging them to focus on both their goals and their physical and mental well-being. Nonetheless, it is essential to acknowledge that the present study did not incorporate externally validated clinical benchmarks or diagnostic cut-off values to delineate the boundaries of the identified EMSs profiles. Consequently, the designation of the “high EMSs” subgroup should be interpreted with caution, particularly regarding its clinical significance and applicability. While the latent profile analysis yielded statistically meaningful classifications, the absence of established clinical thresholds constrains the extent to which these profiles can be translated into clinical practice or diagnostic decision-making. Future investigations would benefit from integrating standardized clinical assessment tools—such as structured diagnostic interviews or validated symptom severity scales—to map EMSs profiles onto clinically recognized categories. Such an approach would enhance the external validity of the typology and support its practical relevance for clinical diagnosis, case formulation, and intervention planning.

### 4.2 Factors influencing the latent categories of EMSs in college students

This study found that gender and grade may influence the latent classifications of EMSs in college students. Women are more likely than men to belong to the high EMSs, which is consistent with previous research (Irkörücü, 2016). Shorey et al. found that the gender differences in EMSs are due to gender role differences (Shorey et al., 2012). For men, societal stereotypes emphasize autonomy, self-interest, and self-protection, but for women, they promote social bonds, community, and the interests of others, with less emphasis on agency and self-development. Women are generally more likely than men to have had traumatic childhood events (Abele and Wojciszke, 2007), which are one of the causes of early maladaptive schema development (Young et al., 2006). However, it is essential to acknowledge that these observed differences may also be influenced by reporting biases and contextual stressors, rather than being attributable solely to developmental factors. Therefore, university mental health education should pay more attention to female students by offering targeted mental health lectures and counseling courses to help them break free from societal stereotypes and enhance their self-awareness. Senior students are more likely than freshman to belong to the high EMSs, which could be because negative cognitive tendencies become more ingrained as people age (Bolen and Scannapieco, 1999), faced with the uncertainty and stress of future life, these schemas are more easily triggered (Pilkington et al., 2021). Future research should consider the role of contextual variables, such as life stressors and societal expectations, which may also influence these differences. To solve this issue, institutions should improve career planning guidance and psychological support for senior students. Systematic career exploration classes should be offered early in university to help students clarify their chosen path and reduce worry caused by future uncertainty. For Senior students, psychological counseling services can be provided to help them adjust their mindset and change negative cognitive patterns, enabling them to cope with pressure in a more positive way. Additionally, cultural factors may influence how EMSs develop and persist. In collectivist cultures like China, values such as emotional restraint, filial piety, and social conformity may reinforce certain schemas, especially those related to self-sacrifice or perfectionism. Future research should explore these cultural influences to better inform targeted interventions.

The study also found that self-compassion, perceived social support, and psychological flexibility predict the latent classifications of EMSs in college students. This indicates that positive psychological resources play a pivotal role in alleviating the detrimental effects of EMSs on mental health. The synergistic effect of self-compassion, perceived social support, and psychological flexibility provides a more nuanced and comprehensive understanding of EMSs profiles, offering an explanation that extends beyond what is accounted for by the structural severity of EMSs alone. This finding emphasizes the critical importance of integrating positive psychological attributes into the EMSs framework, thereby advancing our understanding of how individuals adapt to and cope with maladaptive schemas. As self-compassion scores rise, college students become more likely to belong to the low EMSs. However, given the cross-sectional design of the study, causal conclusions between self-compassion and EMSs cannot be drawn. A longitudinal study conducted with adolescents found that training individuals to accept their own shortcomings and build compassionate and self-kind skills helped minimize social anxiety symptoms during the transition from youth to adulthood (Ştefan, 2019). Moreover, self-compassion has adaptive value, as it can serve as an effective emotional regulation strategy when individuals face stressful life events or situations (Diedrich et al., 2014). Individuals with higher levels of self-compassion are able to reduce negative self-evaluations, avoid excessive self-blame, and alleviate emotional distress, which in turn helps mitigate the emotional problems caused by EMSs (Faustino et al., 2020). Based on this, university mental health education should prioritize self-compassion training programs. Universities can help students accept their flaws, increase their self-compassion, and lessen the detrimental influence of EMSs by providing mindfulness training and other ways.

Perceived social support is concerned with an individual’s cognitive appraisal of their surroundings, as well as their trust in obtaining assistance and support. Its goal is to assess the availability, sufficiency, and insufficiency of various sorts of support. In essence, perceived social support represents a psychological sense of belonging, acceptance, love, and emotional connection (Moghtader and Shamloo, 2019). As perceived social support scores increase, college students are more likely to belong to the low EMSs. By enhancing emotional support and informational support, perceived social support can effectively mitigate the negative impact of early maladaptive schemas (Lorzangeneh and Esazadegan, 2022). It not only improves emotional regulation and strengthens self-worth, but also enhances psychological resilience, reducing the internalization and reinforcement of maladaptive schemas (Hosseini et al., 2016). Universities may improve family-school collaboration, promote a positive campus culture, and encourage students to join student clubs and social activities. These programs give kids with enough emotional and informational support, increase their perceived social support, assist them in reducing the detrimental influence of early maladaptive schemas, and, eventually, improve their self-esteem.

The two primary features of psychological flexibility are experiential avoidance and cognitive fusion. In this study, the Avoidance and Fusion Questionnaire was used, and higher scores imply lower psychological flexibility and greater psychological rigidity (Chen et al., 2019). Therefore, as psychological rigidity increases, it serves as a risk factor for EMSs, making college students more likely to belong to the high EMSs. The theoretical foundation of psychological flexibility lies in executive function and personality structure. Strong executive function allows people to retain high levels of synchronization in their emotions, cognition, and conduct. Individuals with an integrated personality structure can respond well to overall stressors, helping them to better adjust to environmental changes (Kashdan and Rottenberg, 2010). From a psychodynamic perspective, individuals with high psychological flexibility possess more mature psychological defense mechanisms, enabling them to cope with stress in a healthy manner and preventing the solidification of early maladaptive schemas (Altieri et al., 2024). Therefore, universities can help students enhance psychological flexibility by offering cognitive-behavioral training courses and organizing team-building activities. These interventions can reduce psychological rigidity, prevent the reinforcement of EMSs and better equip students to handle the challenges of college life. In conclusion, due to the cross-sectional design, causal relationships cannot be established. Also, the regional sample limits generalizability. Future studies should adopt longitudinal and cross-cultural designs to validate and extend these findings.

## 5 Implications

The findings imply that while performing mental health work in universities, tactics should be tailored to the diverse requirements of students. A complete and multilayered mental health support system can be built by combining the principles of self-compassion, perceived social support, and psychological flexibility. Specific treatments can be implemented via digital tools, and home-school collaboration can be improved by arranging lectures and communication channels to discuss tactics. Encouraging pupils to engage in social behaviors can improve psychological resilience in all dimensions. For senior students, career guidance centers in universities should provide support tailored to their major, internship experiences, and career interests to alleviate anxiety caused by career uncertainty. Moreover, EMSs in college students should be examined from a gender-differentiated perspective. For female students, their emotional sensitivity and communication skills can be leveraged to organize dedicated psychological growth groups to enhance their ability to cope with early maladaptive schemas.

## 6 Limitations and future research directions

For starters, the sample in this study was centered in northwest China, therefore the findings may not fully represent the genuine situation of college students from other geographical regions or colleges of varying tiers. Future study can broaden the sample’s geographical breadth to include places with varying levels of economic development and students from a variety of universities. Second, this study assessed EMSs using self-reported measures, which are vulnerable to subjective bias, and only examined schemas at a single time point from a static perspective, resulting in a lack of long-term tracking of EMSs development in college students. A notable methodological limitation of the present study is that the latent classification was derived from the total EMSs score, rather than from multidimensional schema-domain profiles. While the total score provides a parsimonious index of overall schema severity, this approach may inadvertently obscure clinically meaningful heterogeneity across specific domains. For example, individuals exhibiting elevated scores in Disconnection/Rejection but low levels in Impaired Limits may have been subsumed under the same profile, despite possessing distinct psychological risk profiles and intervention needs. Future study can use longitudinal tracking and measurement of EMSs by semester or academic year to map out the developmental trajectories of these schemas and identify temporal patterns. Moreover, domain-specific latent profile modeling should be adopted to capture more nuanced and differentiated schema patterns, which could improve both theoretical precision and the clinical applicability of interventions. Furthermore, a comprehensive study methodology that includes behavioral observation, neurophysiological measurement, and other approaches can provide multidimensional validation and augment questionnaire results. Finally, this study did not fully consider the impact of socio-cultural factors, and future research can explore this topic in cross-cultural contexts.

## 7 Conclusion

This study employed latent profile analysis to divide college students’ EMSs into three latent profiles: low EMSs; moderate EMSs and high EMSs. Gender, grade, self-compassion, perceived social support, and psychological flexibility all have predictive effects on the classification.

## Data Availability

The raw data supporting the conclusions of this article will be made available by the authors, without undue reservation.
